# High-Throughput Identification of Chemical Inhibitors of *E. coli* Group 2 Capsule Biogenesis as Anti-Virulence Agents

**DOI:** 10.1371/journal.pone.0011642

**Published:** 2010-07-19

**Authors:** Carlos C. Goller, Patrick C. Seed

**Affiliations:** Department of Pediatrics, Center for Microbial Pathogenesis, Duke University, Durham, North Carolina, United States of America; University of California Merced, United States of America

## Abstract

Rising antibiotic resistance among *Escherichia coli*, the leading cause of urinary tract infections (UTIs), has placed a new focus on molecular pathogenesis studies, aiming to identify new therapeutic targets. Anti-virulence agents are attractive as chemotherapeutics to attenuate an organism during disease but not necessarily during benign commensalism, thus decreasing the stress on beneficial microbial communities and lessening the emergence of resistance. We and others have demonstrated that the K antigen capsule of *E. coli* is a preeminent virulence determinant during UTI and more invasive diseases. Components of assembly and export are highly conserved among the major K antigen capsular types associated with UTI-causing *E. coli* and are distinct from the capsule biogenesis machinery of many commensal *E. coli*, making these attractive therapeutic targets. We conducted a screen for anti-capsular small molecules and identified an agent designated “C7” that blocks the production of K1 and K5 capsules, unrelated polysaccharide types among the Group 2–3 capsules. Herein lies proof-of-concept that this screen may be implemented with larger chemical libraries to identify second-generation small-molecule inhibitors of capsule biogenesis. These inhibitors will lead to a better understanding of capsule biogenesis and may represent a new class of therapeutics.

## Introduction

*Escherichia coli* is the leading cause of community-acquired urinary tract infections (UTIs), producing over 80% of community-acquired UTI and at least 50% of nosocomial UTIs [1]. Twenty five to forty percent of first-time community-acquired UTIs are followed by recurrences caused by the same clone of UPEC. In addition, *E. coli* also accounts for a significant proportion of sepsis and meningitis of the young and old, with the infections originating from the urinary tract or direct translocation from the gut into the bloodstream. With over 100 million UTIs occurring annually throughout the world, including more than 10 million cases in U.S. adolescents and adults (per NIDDK data, [2]), UPEC accounts for substantial medical costs and morbidity worldwide.

Accompanying the large use of antibiotics for UTI and other common infections like those of the respiratory tract has been rising antibiotic resistance among *E. coli*, resulting in many cases in multidrug resistant strains [3], [4], [5], [6], [7], and invigorating efforts to elucidate vulnerable targets in the molecular pathogenesis of infection. Of the oral therapies for community-acquired UTI, trimethoprim-sulfamethoxazole (TMP-SMX) has been a mainstay in outpatient therapy, but resistance to TMP-SMX has recently emerged among urinary tract isolates with rates in excess of 20% in some areas (e.g., [8]). The Infectious Diseases Society of America (IDSA) now recommends that in regions where resistance to TMP-SMX exceeds 15%, TMP-SMX should no longer be used for empirical therapy [9]. Ciprofloxacin and other fluoroquinolones are used increasingly, but resistance to these agents is also on the rise (e.g., [3]), and fluoroquinolone-resistant isolates of *E. coli* are often multidrug resistant [10]. A common choice for empiric therapy of acute uncomplicated UTIs is nitrofurantoin; however, resistance to this antibiotic is also increasing [11], and its use requires longer treatment courses while being a poor treatment of outpatients treated for pyelonephritis Much of the resistance arises from bacteria such as *E. coli* that also colonize sites separate from the sites of infection such as the enteric tract. Inhibitors capable of attenuating an organism during disease but not promoting resistance among colonizing bacteria are attractive therapeutics that would bolster the arsenal of failing antibiotics.

One approach to new anti-infectives is to create drugs that render microbes vulnerable to host clearance mechanisms such as innate immunity without being directly detrimental to the target organism. Multiple innate defense mechanisms are thought to participate in clearance of bacteria from the urinary tract. A robust pro-inflammatory cytokine response of IL-6 and IL-8 results from TLR4-LPS stimulation [12], [13], [14], [15]. Subsequently neutrophils are recruited into the urinary tract, producing pyuria. Complement levels increase during inflammatory conditions in the urinary tract [16] and may be an important mechanism of defense. Antimicrobial peptides (AP), including the cationic 3–5 kDa peptides called defesins, are abundant in the urine [17]. AP form pores in phospholipid bilayers but require access to the bacterial outer membrane for function [18]. The effectiveness of the innate immune response against bacteria such as *E. coli* may, however, may be hindered by bacterial factors such polysaccharide capsules. *E. coli* is also a well-recognized cause of urosepsis, and bacteria translocating from the urinary tract into the bloodstream are subject to most of these same assaults as enacted by the innate immune system of the urinary tract.

Capsules are well-established virulence factors for a variety of pathogens and serve to protect the cell from opsonophagocytosis and complement-mediated killing (reviewed in [19], [20]). K capsules, also called K antigens, are enveloping structures composed of acidic, high-molecular-weight polysaccharides. Among UPEC, the K antigens K1, K5, K30, and K92 are most prevalent [21]. In recent work, Llobet *et al.* demonstrated that highly acidic polysaccharide capsules of *K. pneumoniae, P. aeruginosa*, and *S. pneumoniae* interact strongly with APs, acting as “sponges” to sequester and neutralize the APs [22]. Furthermore, we have found that K capsule contributes to multiple aspects of UTI pathogenesis, including intracellular replication [23], making inhibition of capsule biosynthesis a novel target for attenuation of UPEC virulence.

The Group 2 K capsule genes can be divided into three genetic regions: ASSEMBLY (I), SYNTHESIS (II), and EXPORT (III). Group 2 & 3 capsules require homologous ASSEMBLY and EXPORT proteins, but the genes for Group 3 are re-distributed among the Region I-III gene clusters. In addition, many of the components encoded in Regions I and III have homologues in other medically important bacteria such as *Neisseria meningitidis*, and these counterparts also participate in capsule biogenesis. Among the *E. coli* Group 2 capsules, the K1 and K5 serotypes account for the majority of UPEC K clinical isolates. K1 is composed of α2,8 linked poly-Neu5Ac, and K5 contains repeating *N*-acetylglucosamine and glucuronic acid units [24], [25].

Genetic disruption of Group 2 and Group 3 capsule production results in predictable phenotypes, and determining if and where polysaccharide accumulates in the cell can point to where capsule biogenesis is blocked by genetic mutation or chemical inhibition. Interruption of many points in the SYNTHESIS genes abrogates the accumulation of intra- and extracellular polymers and renders the organism insensitive to K1F phage, which requires surface capsule for entry [26], [27].

Inhibiting K capsule production may sensitize the organism to a conventional antibiotic or component of the immune system. Proof-of-concept evidence comes from the demonstration that injection of purified K1 endosialiase prevented sepsis and meningitis after intraperitoneal infection of neonatal rats with *E. coli* K1 [28]. Endosialidase had no direct *in vitro* effect on *E. coli* K1 viability, but presumably removed K1 capsule *in vivo*, rendering the organism more susceptible to host immune factors and external stress, attenuating the infection. However, endosialidases have limited therapeutic applications due to their antigenicity, poor bioavailability, and potential action on sialidated host proteins and lipids with shared linkages as the capsular sialic acids [such as those present in neural tissues, reviewed in [29]]. Furthermore, endosialidase has a very narrow biochemical target range, limiting its application to specific K antigen types. Chemical inhibition of K capsule production may achieve similar therapeutic results without most of these limitations.

Exploiting the properties of K capsule-specific phage, we designed an innovative yet simple screen to uncover small molecule inhibitors of capsule biogenesis. In this report, we highlight the potential of our approach by describing the identification and basic characterization of a novel agent designated “C7”. This agent is active (IC_50_ between 12.5–25 µM), blocks the production of K1 and K5 capsule biogenesis and lacks obvious toxicity to cultured bladder epithelial cells. Beyond the scope of C7 and its analogues as potential capsule inhibitors, the knowledge gained through these studies will expedite future large screens for additional broad spectrum capsule inhibitors, elucidation of their molecular targets, and evaluation as anti-therapeutics.

## Results

### High-throughput screen for small compound inhibitors of capsule

**Figure 1:**
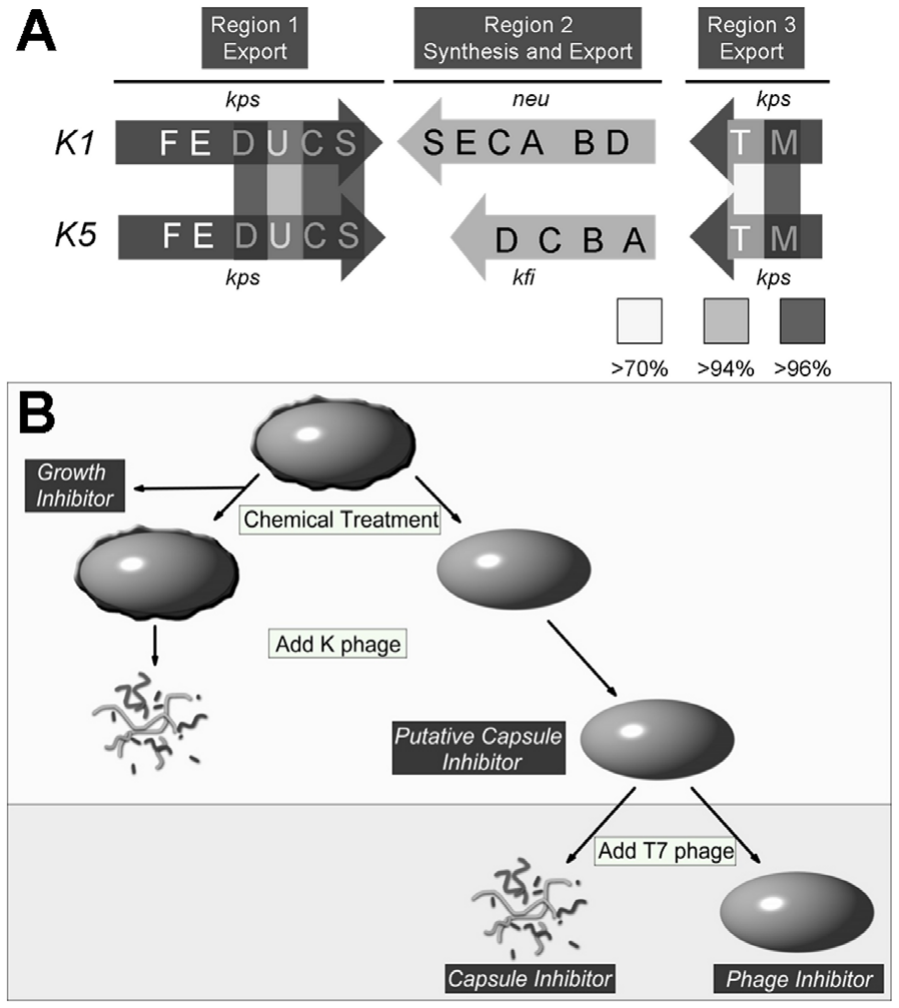
Group 2 capsules and overview of screening strategy to identify capsule biogenesis inhibitors. (**A**) *Organization of the genomic region encoding for major K capsule synthesis and assembly products*. The Group 2 capsules are encoded from similar loci. The SYNTHESIS gene products derive the primary monosaccharides necessary for capsule and render the monosaccharides competent for polymerization. The ASSEMBLY and EXPORT products co-assemble to polymerize the capsule and transport it out of the bacterium where it envelops the cell. As examples, K1 and K5 capsules differ in Region II gene number and identity. Interconnecting lines indicate degree of amino acid identity between key structural components of capsule biogenesis encoded from Region I and Region III. Thick, thin, and doted lines indicate >97%, >94%, and >70% identity, respectively (further details in Table S1). (**B**) *Diagram of high-throughput screen for capsule inhibitors*. Wells of a 96-well plate were seeded with a dilution of UPEC K1 strain UTI89. Compounds from the DTP small molecule library were placed in appropriate wells at a final concentration 100 µM. After a short incubation, the K1 capsule specific K1F phage was added to these wells. Growth was monitored and compounds that did not produce an initial growth inhibition but did inhibit phage lysis, suggesting altered capsule biosynthesis or phage inhibition, were further tested. Secondary assays (indicated by shaded block) were conducted using the engineered K-12:K1 strain EV36. T7 phage, inhibited by T7 capsule but genetically similar to K1F phage, was used to infect this strain. Compounds that sensitized cells to T7 phage were classified as having capsule-specific effect(s) and selected for further analysis. Finally, the K-12 strain MG1655, which is readily lysed by T7 phage, was used in a final assay to determine which compounds inhibited phage lysis.

Based on the conservation between the assembly and export components of Group 2 capsule biogenesis (shown in Figure 1A), we hypothesized that chemical inhibitors of multiple K type capsules could be identified. A high-throughput screen for inhibitors of encapsulation was developed in which the prototypic uropathogenic *Escherichia coli* strain UTI89 was grown in LB broth in 96-well plates in the presence of 100 µM compound or 1% DMSO vehicle followed by treatment with the K1 capsule specific phage K1F (K1F φ). Figure 1B depicts the screening process.

A wild-type UTI89 K1 encapsulated strain grown in the presence of vehicle and treated with K1F phage at an OD_600_≈0.1–0.2 was quickly lysed and served as a positive control. In contrast, a genetic capsule assembly mutant (UTI89 Δ*kpsM*) was resistant to K1F phage lysis and was used as a negative control. Compounds that affected capsule synthesis or assembly were predicted to produce bacterial resistance to phage. A large signal-to-noise ratio and low frequency of false positives make this a very robust assay. The Z' factor, a measurement of the suitability of a particular assay for use in a full-scale high-throughput screen, was determined to be 0.93 when performed in a 96 well microtiter plate format.

We expected one of multiple outcomes from the assay. First, chemical inhibitors of bacterial growth were expected to be present in most chemical libraries. An initial absorbance reading after ∼1 hr of growth was used to determine those compounds that severely affected bacterial growth (arbitrarily defined as those with an OD_600_ less or equal to 0.05 after first time point and no increase thereafter). Compounds producing growth inhibition were not further analyzed. Of the compounds that did not inhibit bacterial growth but prevented phage lysis, we anticipated they would be categorized into two primary groups: 1) those affecting capsule biogenesis (true positive) and 2) those inhibiting the phage lifecycle (false positive). The later groups could be identified and eliminated by the secondary screen described later.

In total, 2,195 compounds from the Developmental Therapeutics Program at the National Cancer Institute were screened. Seventy five (3.41%) of the compounds produced significant inhibition of growth and were eliminated. Thirty five (1.59%) compounds inhibited K1F phage lysis in the 96 well plate format. However, only 9 of these 35 reproducibly inhibited phage lysis in a larger shaken tube format and these 9 were advanced into the secondary screening process. Table 1 summarizes the results of the initial high-throughput screen.

**Table 1:** Summary of high-throughput screen.

	N	%
Total compounds screened	2195	-
Compounds causing growth inhibition	75/2195	3.42
Inhibited K1 lysis	35/2195	1.59
Sensitized K-12:K1 to T7 phage	2/9	0.09
Inhibited T7 lysis of K-12 strain	7/9	0.32

### Secondary assays

Compounds that inhibited K1F phage lysis in the primary assay were further tested in secondary screens to distinguish between inhibition of capsule biogenesis and inhibition of phage replication. The secondary screens capitalize on the knowledge that T7 and K1F phage are genetically and physiologically closely related [27]. However, K1F phage entry requires K1 capsule, while T7 phage entry is inhibited by the capsule. As a result, only unencapsulated bacteria are subject to T7 infection and lysis [30].

The engineered K12:K1 hybrid strain called EV36 producing K1 capsule was grown in the presence of the putative capsule inhibitors and was then infected with T7 phage (T7φ).

Compounds that sensitized the K12:K1 strain to T7 phage were deemed to affect capsule, and 2 of 9 compounds rendered EV36 sensitive to T7. The confirmation that these 2 compounds were effective in eliminating capsule in this well-characterized K1 hybrid strain is that it confirmed the capsule inhibition effects in an independent unrelated genetic background (Table 1).

To further confirm that the effect of each compound was not due to inhibition of phage replication, the prototypic K-12 strain MG1655, which lacks K1 capsule and is readily susceptible to T7 phage infection, was grown in the respective compounds. Compounds inhibiting T7-mediated lysis of MG1655 were excluded as being phage-specific and not affecting capsule biogenesis. Several compounds inhibited T7 lysis of MG1655, indicating that they likely affected phage physiology (Table 1). Table 2 lists the agents that passed the primary screen and indicates the performance of each chemical in the secondary assays.

**Table 2:** Summary of capsule-specific and phage specific effects of selected lead compounds.

NSC number	K1F phage inhibition	EV36/T7 phage lysis	MG/T7 phage lysis	Interpretation
35605	Yes	No	No	Phage specific
13028	Yes	No	Yes	Phage specific
136469 (C7)	Yes	Yes	Yes	Capsule specific
5550	Yes	Yes	Yes	Capsule specific
5159	Yes	No	Not for 2 hr	Phage specific
661755	Yes	No	Not for 2 hr	Phage specific
322921	Yes	No	No	Phage specific
311153	Yes	No	Not for 2 hr	Phage specific
311152	Yes	No	Not for 3 hr	Phage specific

### C7 as lead compound

**Figure 2:**
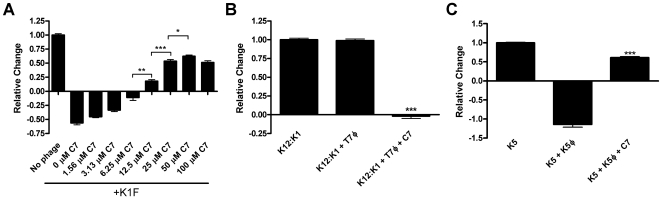
C7 inhibition of K1 and K5 capsule dependent phage lysis. (**A**) C7 inhibits K1 capsule-dependent phage lysis of UPEC. UPEC K1 strain UTI89 was grown in the presence of 1% DMSO or indicated concentrations of C7 for 1 hr and K1F phage was added where indicated. Relative OD_600_ change after 4 hrs is shown. Increasing C7 concentrations inhibit lysis of UPEC K1. P value: <0.0001 No C7 (0 µM) compared to each of C7 treatments >12.5 µM. (**B**) C7 sensitizes K12:K1 strain to T7 phage. The K12 strain EV36 producing K1 capsule was infected with T7 phage (T7φ), which is inhibited by the presence of K1 capsule. Addition of 100 µM C7 sensitized EV36 to T7 phage (p = <0.0001). **(C)** C7 inhibits K5 capsule-dependent phage lysis. UPEC K5 strain DS17 was incubated with (K5φ) or without phage and relative change in OD_600_ was recorded. Addition of 100 µM C7 prevented lysis by K5 capsule specific phage (p<0.0001).

In our initial screen, we identified 2 inhibitors of capsule biogenesis (Table 2). Although not previously described as an inhibitor of capsule biogenesis, NSC5550, also known as malachite green oxalate, produces metabolites with known toxicities to mammalian systems and was therefore not pursued further at this time. The other inhibitor, 2-(4-phenylphenyl)benzo[g]quinoline-4-carboxylic acid (NSC136469), was investigated further. We designated this molecule as “C7” and pursued it as a prototype for a chemical inhibitor of capsule biogenesis. C7 reproducibly inhibited K1F phage lysis of UPEC K1 strain UTI89 in tests following the high throughput screen (Figure 2A), and the inhibition was found to be dose-dependent with the effect reaching saturation at ∼25 µM C7 (p = 0.3731 25 µM and 100 µM C7), producing ∼50% inhibition of K1F phage lysis of UPEC at 12.5–25 µM (Figure 2A). C7 activity was also tested on the K12:K1 strain EV36, a reciprocal experiment of the K1F lysis inhibition. EV36 grown in the presence of 100 µM C7 was highly susceptible to T7 phage, suggesting that C7 inhibited K1 capsule production and allowed T7 to attach, enter, and lyse the target bacteria (Figure 2B). Next, we determined if C7 could inhibit a distinct non-K1 Group 2 K capsule-expressing strain. The UPEC K5 pyelonephritis isolate DS17 was grown in the presence and absence of C7. Without C7, DS17 was readily lysed by K5 bacteriophage. In contrast, treatment with 100 µM C7 nearly completely inhibited K5 bacteriophage lysis (Figure 2C). In summary, the phage data suggests that C7 acts also on K5 capsule production/assembly and that C7 targets a convergent point in the production of these two different Group 2 polysaccharide capsules. These data provide proof-of-principle that this simple and efficient high-throughput screen of a relatively limited compound library is able to yield candidate small molecule inhibitors of Group 2 capsule biogenesis.

### Effect of C7 on capsule

**Figure 3:**
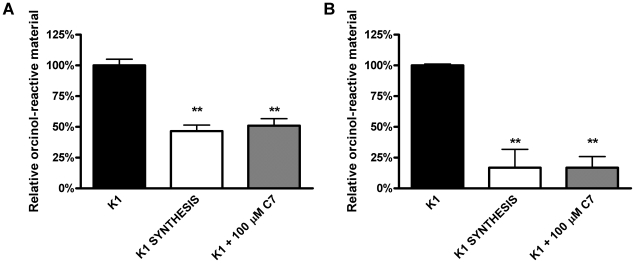
C7 treated cells produce less orcinol-reactive carbohydrates. Cells were (**A**) sonicated or (**B**) treated with mild acid to release cell surface polysaccharides. Material was then extracted and polysaccharides were detected using the orcinol reagent. C7 treated cells had levels of orcinol-reactive carbohydrates comparable to those of a capsule synthesis (K1 SYNTHESIS) mutant for both whole cell sonicates as well as released surface material. This suggests that capsule levels are reduced by C7 treatment, and the compound does not simply release capsule from the bacterial surface. Absorbance readings were expressed as the percent reactivity of the wild-type UTI89 K1 strain. K1 vs. SYNTHESIS mutant or C7 treated, p<0.01; SYNTHESIS mutant vs. C7 treated p>0.05 by Tukey's t-test for both (**A**) and **(B)**.

Based on the results of our phage assays, we hypothesized that C7 acts on a step in capsule assembly or export that is common to both K1 and K5 capsule types. In order to localize the point of inhibition and the molecular target of C7, we sought to determine the phenotypic consequences of C7 treatment on capsule biogenesis, and whether C7 treatment resulted in capsule release, intracellular accumulation of polymer, or inhibition of synthesis. Whole cells and sonicates were used in agglutination and radial immunodiffusion assays using the H46 anti-K1 capsule polyclonal antiserum. Whereas wild-type and genetic capsule synthesis mutants were positive and negative for agglutination, respectively, C7 treated UTI89 did not agglutinate, and whole cell sonicates had only weak reactivity (Table 3). We next sought biochemical evidence that C7 inhibited capsule production in UPEC K1. Extracted polysaccharides obtained from whole cell sonicates or surface capsule released (by mild acid treatment (adapted from [31]), were treated with Bial's orcinol reagent that reacts with pentoses [32]. Polysaccharides extracted from whole cell sonicates of C7 treated cells yielded significantly lower orcinol reactivity than extracts from untreated cells (p<0.01), but without statistical differences in reactivity compared to extracts from a genetic SYNTHESIS mutant (Figure 3A). Consistent with the whole cell data, acid-released polysaccharides from C7 treated cells also had low orcinol reactivity, statistically no different than a SYNTHESIS mutant (UTI89 Δ*neu*; Figure 3B). We tested if C7-treatment resulted in spontaneous release of polysaccharide into the media without proper anchoring into the outer membrane. LPS alterations have previously been shown to affect encapsulation [33]. However, concentrated ultrafiltrates of culture supernatants from C7-treated bacteria did not produce agglutination with antibody (data not shown). Furthermore, C7 treatment did not affect LPS abundance or migration on SDS PAGE gels (Figure S1; method described in Materials and References S1), indicating that lack of phage sensitivity and other phenotypes associated with C7 treatment are not due to defects in LPS. Together, these results suggest that C7 may be blocking an early step in capsule assembly, since weak agglutination and orcinol reactivity of whole cell sonicates of C7-treated cells indicates little to no intracellular accumulation of capsule and there was no detectable release of capsule upon acid treatment.

**Table 3:** Summary of agglutination assays.

Strain	Whole cell sonicates	Radial immunodiffusion assay
K1	+	+
K1 synthesis	−	−
K1+100 µM C7	weak	−

Whole cells and sonicates were used in agglutination and radial immuno diffusion assays using the H46 anti-K1 capsule polyclonal antiserum. Whereas wild-type and capsule synthesis mutants were positive and negative for agglutination, respectively, C7 treated UTI89 did not agglutinate and whole cell sonicates had only weak reactivity.

Whole cells and sonicates were used in agglutination and radial immuno diffusion assays using the H46 anti-K1 capsule polyclonal antiserum. Whereas wild-type and capsule synthesis mutants were positive and negative for agglutination, respectively, C7 treated UTI89 did not agglutinate and whole cell sonicates had only weak reactivity.We predicted that an inhibitor of K1 and K5 capsule production was unlikely to target monosaccharide synthesis since the compositions of the K1 and K5 capsules are different, thus lacking an obvious central target of inhibition in early synthesis shared between both types. To confirm that C7 did not inhibit sialic acid synthesis and thus K1 capsule production, cultures were grown in the presence of up to 500 µM of N-acetyl neuraminic acid (NANA; sialic acid). However, this supplementation to the medium did not restore sensitivity of C7 treated strains to K1F phage lysis, suggesting that K1 capsule was not produced (data not shown). These data suggest that inhibition is not directly on the components of the synthesis pathway, since prior studies have shown that defects in NeuB or NeuC, critical synthesis enzymes encoded in Region II (Figure 1A), can be corrected by addition of exogenous precursor sialic acid [34], [35].

### C7 analogues as capsule inhibitors

In order to gain more insights into the important structural elements of C7, we also tested a limited series of structurally related compounds for inhibition of K1F phage lysis (Figure S2); however, none of the compounds tested was highly active. NSC201538 (2-(4-dimethylaminophenyl)benzo[g]quinoline-4-carboxylic acid) had limited activity, where the major substitution differentiating it from C7 was in the 4-dimethylaminophenyl substitution in place of the 4-phenyl-phenyl group present in C7, demonstrating the importance of the specific R-group for C7 activity.

### Treatment increases C3 binding to UPEC K1 and serum sensitivity

**Figure 4:**
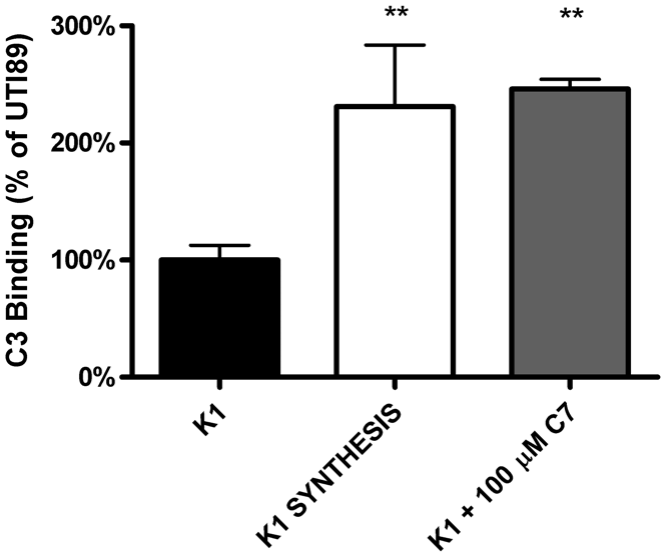
C7 increases C3 binding to UPEC K1. UTI89 was incubated with human serum and C3 binding was quantified by densitometry of immuno-dot blot. C7 treatment increases C3 binding to levels similar to an unencapsulated cell (K1 SYNTHESIS mutant; p = 0.0054).

As noted previously, polysaccharide capsules have known inhibition effects on complement binding to bacteria. We hypothesized that C7 treatment of UPEC K1 would result in increased binding by complement C3, initiating recruitment of the complement attack complex. UPEC K1 was treated with C7 or vehicle control and then incubated in normal human serum. C7 treatment produced a significant increase in C3 binding to the treated bacteria (∼2-fold, Figure 4). These data suggest that C7 treatment renders the cells more susceptible to C3 binding, which in turn is known (specifically through C3b) to promote formation of the MAC complex and phagocytosis.

**Figure 5:**
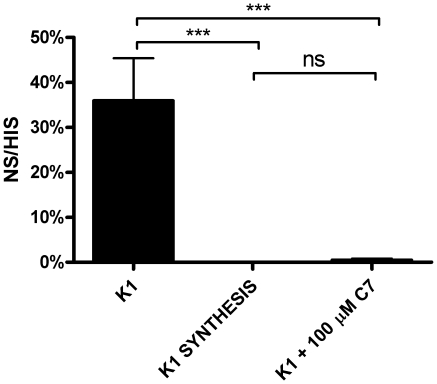
C7 sensitizes K1 encapsulated UPEC to human serum. Three ml cultures of the K1 encapsulated UPEC strain UTI89, a capsule synthesis mutant (K1 SYNTHESIS), and wild-type cells treated with 100 µM C7 were grown in LB +1% DMSO or C7 for ∼3 hrs with shaking until reaching an OD_600_ of 0.8. Human serum from at least two individuals was heat inactivated (HIS) at 55°C for 45 min or maintained on ice (Normal Serum, NS). Bacterial cells were resuspended in PBS, diluted to 5–9×10^3^ and incubated in RPMI/20% serum supplemented with 1% DMSO or 100 µM C7 at 37°C for 2.0 hrs. Duplicate independent cultures for each strain were tested and the ratio of NS/HIS CFU/ml is shown. The graph represents the average of two experiments.

Given the increased binding of C3 to C7-treated cells, we anticipated that the chemically treated cells would have increased serum sensitivity similar to a genetic capsule mutant. Because of the established role of capsule in resistance by *E. coli* to serum killing, chemical capsule inhibition would be expected to sensitize encapsulated strains to serum exposure, thus attenuating infection and providing a useful therapeutic intervention. We tested whether exposure of the K1 encapsulated strain UTI89 to C7 increased its sensitivity to human serum in an *in vitro* assay with pooled human serum. After a 2 hour exposure of 10^3^ CFU to 20% normal active human serum resulted, 35% of UPEC K1 remained viable. In contrast, the same serum exposure resulted in complete killing of the SYNTHESIS mutant (Figure 5). C7 treatment (100 µM) of UPEC K1 followed by the same serum exposure resulted in <1% of the bacteria remaining viable, statistically the same as for the genetic capsule mutant (no significant difference by Tukey's Multiple Comparison Test). C7 treatment rendered the cells significantly more serum sensitive than untreated wild type (p<0.001). These results illustrate the potential of capsule biogenesis inhibitors to increase the susceptibility of UPEC to the innate immune response.

### C7 is active on clinical *E. coli* isolates

**Figure 6:**
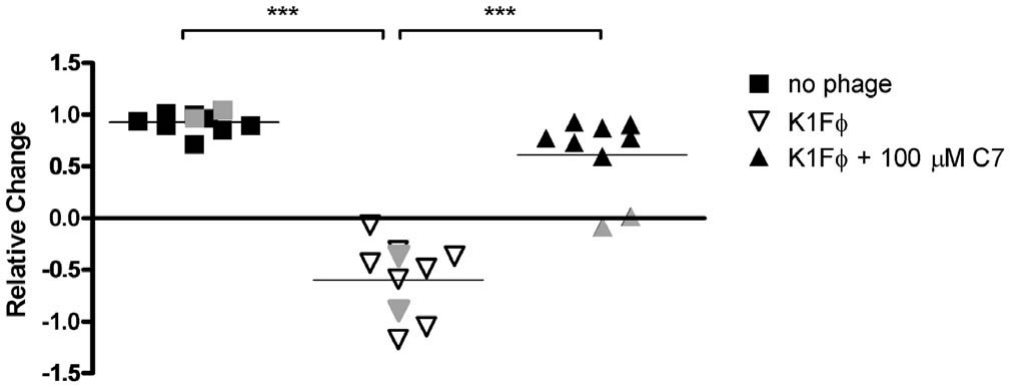
C7 is active against other clinical K1 UPEC strains. A panel of K1F phage sensitive clinical *E. coli* strains was tested for phage sensitivity in the presence or absence of 100 µM C7. Strains were grown in 1% DMSO or C7 and infected with K1F phage where indicated. Relative OD_600_ change after 3 hrs post infection (normalized to the growth of UPEC strain UTI89) is shown. Addition of 100 µM C7 inhibits lysis of tested clinical strains. P value: <0.0001 no C7 (phage only) compared to C7 treatment. Arrow indicates two clinical strains in which C7 only partially restored growth.

To demonstrate that the effect of C7 is not limited to the prototypic laboratory UPEC strain UTI89, capsule inhibition by C7 was tested on a panel of clinical *E. coli* K1 isolates in the K1F phage sensitivity assay. These strains encompass isolates from pyelonephritis (4), recurrent UTIs (3), and single UTI cases (1). As seen in Figure 6, the majority of phage-sensitive isolates tested responded to C7 treatment by becoming insensitive to K1F phage, thus suggesting that capsule production was inhibited by the addition of C7 in these isolates, similar to the situation with strain UTI89. Two strains responded to C7 with only partial insensitivity to K1F, suggesting incomplete inhibition of capsule biogenesis. These results support the idea that C7 is active on a range of clinically relevant strains causing UTIs.

### C7 is non-toxic to bladder epithelial cells

To further assess the therapeutic potential of C7, we next examined the potential toxicity of C7 using a widely used LDH release assay after treatment of the bladder epithelial cell line 5637 at several C7 concentrations up to 100 µM (>4-fold over the IC_50_), as compared to 1% DMSO vehicle. No significant increase in LDH was measured at the maximum tested concentration of C7 compared to the vehicle control (Figure S3; methods described in Materials and References S1).

## Discussion

*E. coli* is by-far the leading cause of community-acquired UTIs. On an annual basis, over 100 million UTIs occur throughout the world with over 10 million infections in the US alone (per NIDDK data, [2]). Rising resistance rates have threatened the arsenal of antibiotics available for the treatment of community-acquired UTI. New therapeutics for UTI are in great demand. The most desirable chemotherapeutics may be those that attenuate an organism during an infection without altering commensal populations, so called anti-virulence agents [36], [37].

UTI involved UPEC cycling between extracellular and intracellular environments. Bacteria initially adhere to the bladder epithelium, following which they may invade the epithelium, and in some cases, escape into the cytosol of the infected epithelial cells and amass into intracellular bacterial communities (IBCs). IBC confer resistance to infiltration by neutrophils [38], [39] and may similarly reduce susceptibility to antibiotic therapy [40]. Intracellular bacteria can emerge from IBCs and cAMP-regulated exocytic vesicles and reinitiate adherence and invasion events [41], [42]. Early in the infection, TLR4 stimulation by LPS [43] results in a strong inflammatory response, neutrophil recruitment, and elaboration of antimicrobial peptides [44], [45]. K capsule is important for UPEC survival in the urinary tract, promoting virulence at multiple steps of pathogenesis, including extracellular and intracellular stages of infection [46]. We recently demonstrated a novel role for the intracellular expression of a polysaccharide capsule as an aggregating factor in IBC formation [46]. Thus, inhibitors of K capsule biogenesis may attenuate the bacterium at multiple stages of infection.

Of the K type capsules, Groups 2 and 3 are overrepresented among the extraintestinal pathogenic *E. coli* including UTI, bloodstream and meningitis isolates. These capsular groups are uncommon among commensal *E. coli*, and therefore, it may be possible to selectively target the pathogenic organisms, particularly during active infection, while leaving commensal organisms unperturbed. In addition to avoiding dysbioses such as antibiotic-induced diarrhea caused by non-specific anti-microbial agents, anti-infectives that do not stress commensal microbial reservoirs may also lessen the emergence of drug resistance. Key components of the assembly and export of Group 2 and Group 3 capsules are highly conserved (such as KpsD, KpsC, KpsU, KpsS, and KpsM), making it theoretically possible to identify small molecules that are capable of inhibiting the biogenesis of a variety of capsules that differ significantly in composition and antigenicity. However, many of these same components are not well conserved among the other capsular types more commonly expressed by commensal strains.

By exploiting the features of capsule-specific phage, we devised an innovative, yet simple screen for small molecule inhibitors of K capsule biogenesis. To demonstrate the throughput and efficiency of the primary and secondary screens to identify capsule biogenesis inhibitors and eliminate false positive hits, we applied the screening procedure to a small collection of molecule libraries and identified one agent designated as “C7” that inhibits the production K1 and K5 capsules, unrelated polysaccharide types among the Group 2 capsules. C7 rendered clinical isolates of K1 and K5 encapsulated UPEC resistant to capsule-specific phage, decreased K1-antiserum agglutination, and shifted the carbohydrate profile of treated cells toward that of a genetic capsule synthesis mutant. Of biological significance, treatment of *E. coli* with C7 rendered the organism more susceptible to C3 binding and serum killing, again mirroring the phenotype of an unencapsulated genetic mutant. The IC_50_ of C7 between 12.5 and 25 µM suggests reasonable activity and a starting point for additional workaround chemistry. The relative hydrophobicity of C7 imposes some limitations on its potential distribution and bioavailability if taken as a drug. Despite any potential limitations of C7, our results provide proof-of-concept that the screening algorithm may uncover additional novel small molecule inhibitors of capsule biogenesis by applying the screen to libraries several orders of magnitude larger in size.

In addition to their potential utility as therapeutic agents to combat infections, chemical inhibitors of capsule biogenesis may provide opportunities to further dissect the molecular processes involved in capsule biogenesis. Our current biochemical and immunological studies of the C7 effects on K1 capsule biogenesis have localized the inhibition to an early stage of capsule assembly, likely after sialic acid synthesis but prior to significant oligimerization of the monosaccharides. Our studies excluded other plausible mechanisms including alteration of LPS and failure to anchor fully polymerized polysaccharide to the bacterial surface. Additional studies will be required to improve our understanding of the molecular mechanisms and targets behind the action of C7. The genetic identity of the target of C7 would provide a better understanding of the capsule biosynthesis process and could greatly enhance the search for chemotherapeutics that could target highly conserved components of the capsule assembly machinery. This could lead to the attenuation of diverse encapsulated organisms with commonalities in capsule assembly. We anticipate that a large chemical library screen currently underway that employs the primary and secondary screens described in this work will yield a variety of active, bioavailable, and synthetically amenable lead compounds to further build on this technology.

## Methods

### Ethics statement

Normal human serum samples were obtained under a protocol reviewed and approved by the Duke University Institutional Review Board. The pooled serum used in these studies was de-identified and anonymous.

### Bacterial strains, phage, and growth conditions

All *E. coli* strains and phage used in the present study are listed in Table 4. Clinical *E. coli* isolates were obtained from Dr. Walt Stamm at the University of Washington. Unless indicated otherwise, bacteria were routinely grown at 37°C in Luria-Bertani medium (LB) with shaking at 250 rpm. Media were supplemented with antibiotics as needed at the following final concentrations: ampicillin, 100 µg/ml; chloramphenicol, 20 µg/ml; kanamycin, 50 µg/ml. LB was supplemented with 1% dimethyl sulfoxide (DMSO; Acros) with or without compound. Phage lysates were prepared from 5 ml cultures of UTI89 (for K1F phage), MG1655 (for T7 phage) or DS17 (for K5 phage) and stored at 4°C over several drops of chloroform as described by [47].

**Table 4:** Bacteria and phage used in study.

Strain/phage	Description or relevant genotype	Reference
*Bacterial strains*		
UTI89	K1 *Escherichia coli* cystitis isolate	[50]
UTI89 Δ*neu*	Region II K1 capsule synthesis mutant	[46]
UTI89 Δ*kpsM*	Region III K1 capsule assembly mutant	This study
DS17	*Escherichia coli* K5 pyelonephritis isolate	[51]
EV36	K12/K1 hybrid produced by conjugation with an Hfr *kps* ^+^ strain; K1 encapsulated, susceptible to K1 specific phage.	[52]
*Phage*		
T7 phage (T7φ)	Inhibited by K1 capsule	[30]
K1F phage (K1Fφ)	K1 capsule specific	[26], [27]
K5 phage (K5φ)	K5 capsule specific	[53]

### Chemicals

Chemical compounds were obtained from the National Cancer Institute's Developmental Therapeutics Program (DTP). Compounds were received in DMSO at a concentration of 10 mM and stored at −20°C in small aliquots protected from light.

### Screen to identify small molecular inhibitors of K1 capsule biogenesis in UPEC

Three ml overnight culture of K1 strain UTI89 grown in LB was diluted 1∶500, and 100 µl per well was added to a 96-well plate (Whatman Uniplate). One microliter of each compound from the DTP small molecule library was placed in appropriate wells (final concentration 100 µM). The first and last columns of the plates received DMSO vehicle alone and were used as controls. Plates were sealed, shaken at 37°C for 2 hr, and the OD_600_ was measured in a μQuant (BioTek) plate reader. Then, 30 µl of a K1F phage lysate was added to each well, except those in the last column, which received LB alone. The plate was re-sealed and shaken for an additional 2 hours before recording the OD_600_ again (“post-infection” measurements). Finally, plates were resealed and shaken overnight for one last growth measurement (“late post-infection”). Assays were repeated twice. Compounds that did not inhibit growth of the test strain prior to the addition of phage but did inhibit phage lysis were further tested in phage assays in test tubes as putative capsule biogenesis inhibitors. Z' was calculated using a previously published equation [48].

### Capsule inhibition assay in test tubes

Overnight bacterial cultures were diluted 1∶100 into three ml of fresh LB supplemented with 100 µM of compound C7 or 1% vehicle control (DMSO). Cultures were grown at 37°C to an optical density OD_600_ of ∼0.2 before addition of appropriate K1 capsule-specific phage or T7 phage (3 µl of lysate). Cultures were incubated further, and growth was monitored by optical density. The initial absorbance before infection was subtracted from the reading at the indicated time point after infection. The log of the absorbance values was then normalized to that of the culture with no phage added, and relative change was plotted. The average of triplicate replicates for a representative experiment was plotted with standard deviations. Each experiment was repeated at least twice with similar results to those shown.

### K1 capsule agglutination assay

Three ml cultures were grown in the presence of 100 µM compound or vehicle to an optical density of ∼0.8, and cells were harvested, washed with PBS, and resuspended in 0.5 ml of PBS. Fiftyµl of cells and 5 µl of undiluted H46 horse anti-Group B *Neisseria meningitidis* capsule (antigenically identical to K1 capsule) polyclonal antiserum [49] were combined, and agglutination was monitored. This assay was repeated at least three times for each strain.

### Radial immunodiffusion assay

PBS plates with 1% agarose were supplemented with 5% H46 antiserum. Cultures grown as for agglutination assays in the presence of 100 µM compound or vehicle were extensively sonicated to produce whole-cell lysates and added to wells created in plates (50 µl). Plates were incubated upright at 30°C for 48–72 hrs and were visually inspected for the formation of precipitin rings.

### Orcinol reaction for carbohydrates

Three ml of cultures of the indicated strains were harvested at OD_600_ = 0.8. For whole-cell orcinol measurements, cells were washed once with PBS, resuspended in 1 ml of PBS, and sonicated. Five hundred microliters of each sonicate were then extracted with phenol:chloroform, and 100 µl was used for labeling. For released material measurements, polysaccharides were separated from the cells by mild acid release (Tris-acetate pH 5.0 for 2 hr with shaking) followed by centrifugation to pellet the cells. Polysaccharides in the cell-free supernatants were then de-proteinated by phenol:chloroform extraction. The aqueous material was concentrated to 50 µl using a YM-30 size exclusion filter.

For labeling, polysaccharides were then hydrolized with 0.1 M HCl for 5 minutes at 90°C in 50 µl of orcinol reagent [32]. Color change was measured by absorbance and expressed as the percentage of wild type levels. Each orcinol reaction was performed in duplicate with independent cultures, and the entire assay was repeated at least twice. A representative experiment is shown.

### C3 binding assay

Human serum from at least two individuals was heat inactivated (HIS) at 55°C for 45 min or was maintained on ice (normal serum, NS). Cells were grown to OD_600_≈1.0 and washed with PBS as described for the agglutination assay. Cells were then pelleted at 8000×g for 1 min and resuspended in DMEM (Sigma) +5% NS or HIS and were incubated at 37°C for 15 minutes. Bacteria were then pelleted and washed three times with PBS before resuspending in 500 µl of PBS. Three microliters were then spotted onto nitrocellulose membranes and allowed to air dry overnight. The membrane was blocked with 5% non-fat dry milk in TBS/0.1% Tween 20 (TBS-T) for 2 hrs, rinsed with TBS-T two times for 15 minutes and exposed to the primary C3 antibody (1∶10,000 in 1% BSA/TBS-T, anti-human C3 developed in goat, from Sigma). After washing two times with TBS-T, secondary anti-goat alkaline phosphatase antibody was applied at a 1∶10,000 dilution. The immuno dot blot was developed using a colorimetric substrate (ImmunoO; MP Biochemicals), and densitometry was performed using Image J (NCBI) software.

### Serum resistance assays

Overnight cultures of the indicated strains were diluted 1∶100 into three ml of LB with 1% DMSO (final) or 100 µM C7. Cultures were grown for ∼3 hrs with shaking until reaching an OD_600_ of 0.8. Bacterial cells were resuspended in PBS, diluted to 5–9×10^3^ CFU/ml and incubated in serum-free RPMI (Sigma) supplemented with 20% human serum and 1% DMSO or 100 µM C7. Cells were incubated in RPMI/serum at 37°C for 2.0 hrs. Duplicate independent cultures for each strain were tested, and the ratio of NS/HIS CFU/ml is shown. Experiments were repeated at least two times.

### Phage sensitivity assays for clinical *E. coli* strains

Clinical *E. coli* strains were screened for K1F and K5 phage sensitivity in 96-well trays. Briefly, a panel of clinical fecal, bloodstream, and urinary tract *E. coli* isolates from Walt Stamm at the University of Washington was arrayed into a 96-well plate and grown with vigorous shaking in LB in deep-well 96-well plates. Cultures were then diluted 1∶100 into 100 µl of LB with 1% DMSO. The plate was sealed with breathable tape and incubated at 37°C with vigorous shaking for 2 hrs (OD_600_ ∼0.1–0.2). Five microliters of a high-titer phage lysate were then added to each well. Growth was monitored spectrophotometrically every 1.5 hrs and “phage sensitive strains” were determined to be those strains for which absorbance decreased after 3.0 hrs of incubation. Each strain was tested in at least four independent plates. Isolates that were consistently lysed by addition of phage were then used in subsequent experiments to determine if addition of 100 µM C7 inhibited phage lysis using the same 96-well plate format. Inhibition of phage lysis by C7 was considered to be growth equivalent to uninfected parent strain after 3 hrs incubation post-infection, and this experiment was repeated at least 4 times with similar results. Relative change in OD was calculated as the log of the absorbance values and normalized to that of strain UTI89 in the same assay. The average of three independent cultures for each strain is shown.

### Statistical analyses

Results were calculated as averages and standard deviations of the means using the Graph Pad Prism 4 software package (San Diego, CA). Nonparametric t-tests were used for statistical analysis of data and calculation of p-values using Graph Pad Prism 4 or Graph Pad online calculators. Significant differences were highlighted with a single asterisk when the P value is less than 0.05, with two asterisks when the P value is less than 0.01, and three asterisks when the P value is less than 0.001.

## Supporting Information

Materials and References S1 Supplemental methods and references.
(0.04 MB DOC)

Figure S1 C7 is non-toxic to bladder epithelial cells. LDH release assay after incubation of 5637 cells with vehicle (1% DMSO) or C7. Concentrations of C7 up to 100 µM did not significantly affect LDH release compared to vehicle control. Triton X detergent control represents maximum LDH release.
(0.16 MB TIF)

Figure S2 C7 treatment does not affect LPS profile. No significant difference was observed in LPS migration or accumulation with and without C7 treatment (Lane 1 vs. 2). The figure represents two independent gels with the same set of samples.
(3.89 MB TIF)

Figure S3 Compounds similar to C7 do not inhibit K1 capsule-dependent phage lysis. *Panel A*: Analogues of C7 tested as K1 capsule biogenesis inhibitors. *Panel B*: K1F phage sensitivity assays with 100 µM C7 and analogues.
(0.98 MB TIF)

Table S1 Similarity and identity of key proteins of group 2 capsule biogenesis in *E. coli*. UTI89 (K1, Group 2 capsule) proteins were compared to other *E. coli* Group 2 capsule homologues and minimum percent identity and similarity indicated. The Basic Local Alignment Search Tool (BLAST, NCBI) was used to compare key Group 2 capsule assembly proteins from the prototypic K1 strain UTI89 with sequenced *E. coli* genomes (taxonomic ID 562) with Group 2 capsule gene arrangement. The following sequenced *Escherichia coli* genomes were considered in the BLAST search: UTI89, SMS 3-5, ED1a, IAI39, APEC01, S88, 042, F11, SE15, BL21 (DE3), Nissle 1917, 101-1.
(0.03 MB DOC)
