# Supplementary material for: High-Throughput Identification of Chemical Inhibitors of E. coli Group 2 Capsule Biogenesis as Anti-Virulence Agents
Source: PLoS One. 2010 Jul 19;5(7):e11642. doi: 10.1371/journal.pone.0011642 (PMC2906519; doi:10.1371/journal.pone.0011642)
Supplement: Materials and References S1 — Supplemental methods and references. (0.04 MB DOC) [file pone.0011642.s001.doc]

**Goller and Seed: Supplemental Methods and References.**

**LDH Cytotoxicity Assay.** Bladder 5636 epithelial cells were seeded in tissue culture treated 96-well plate. The LDH Cytotoxicity Detection Kit (Takara Bio Inc. Kit) was then used to determine cytotoxicity of C7 according to the manufacturer’s guidelines. Briefly, media was replaced with vehicle (1% DMSO) or C7 concentrations up to 100 μM in serum-free DMEM. Triton X-100 detergent (2%) was used as a positive control for maximum LDH release. Cells were incubated for 4 hrs, and LDH release was measured in the supernatant by absorbance.

**LPS isolation and electrophoretic separation.** Three ml cultures were grown in LB with 1% DMSO or 100 μM C7 to OD_600_ = 0.8. Cells were pelleted, washed once with PBS, and resuspended in 0.5 ml of PBS and stored at -20⁰C. 200 μl were pelleted, resuspended in lysis buffer containing 2% SDS, 4% 2-mercaptoethanol, 10% glycerol, 1 M Tris (pH 6.8), and bromphenol blue. Eight μl were then run on a 30% polyacrylamide gel containing 990 mM Tris pH 8.45, 10% glycerol, 1 mg/ml SDS. The anode buffer consisted of 0.2 M Tris and the cathode buffer contained 0.1M Tris, 0.1 M Tricine, and 0.1% SDS. LPS samples were run at 30 volts for 16-18 hrs. The gel was silver stained using a protocol adapted from (Hitchcock & Brown, 1983). Briefly, the gel was rinsed in fixing solution for fifteen minutes (40% methanol, 5% formalin), washed twice with water for five minutes each time, incubated in pre-treat solution (0.02% sodium thiosulfate) for one minute, washed with water for twenty seconds twice, and incubated in 1% AgNO_3_ for fifteen minutes. The gel was then washed twice in water for twenty seconds, before adding developer (3% sodium carbonate, 0.0004% sodium thiosulfate, 0.05% formalin). Bands were allowed to develop for 10-15 minutes before stopping the reaction with one ml of acetic acid. The gel was rinsed in water, dried, and photographed.

**Supplemental References.**

Hitchcock, P. J. & T. M. Brown, (1983) Morphological heterogeneity among *Salmonella* lipopolysaccharide chemotypes in silver-stained polyacrylamide gels. *J. Bacteriol.* **154**: 269-277.
