# Supplementary figures and images for: High-Throughput Identification of Chemical Inhibitors of E. coli Group 2 Capsule Biogenesis as Anti-Virulence Agents
Source: PLoS One. 2010 Jul 19;5(7):e11642. doi: 10.1371/journal.pone.0011642 (PMC2906519; doi:10.1371/journal.pone.0011642)

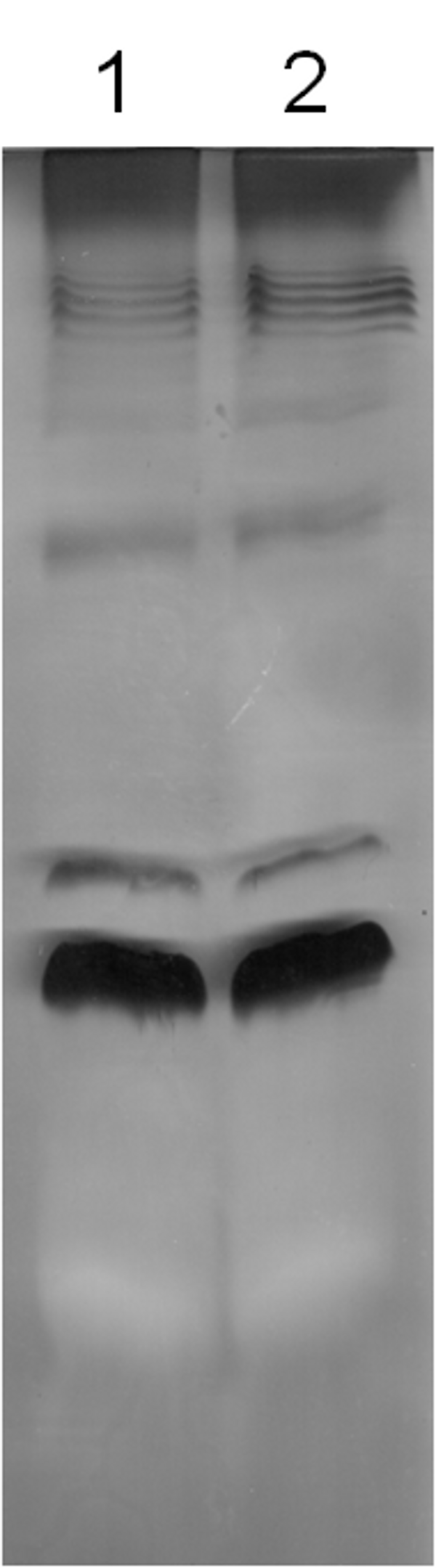

Supplement: Figure S2 — C7 treatment does not affect LPS profile. No significant difference was observed in LPS migration or accumulation with and without C7 treatment (Lane 1 vs. 2). The figure represents two independent gels with the same set of samples. (3.89 MB TIF) [file pone.0011642.s003.tif]

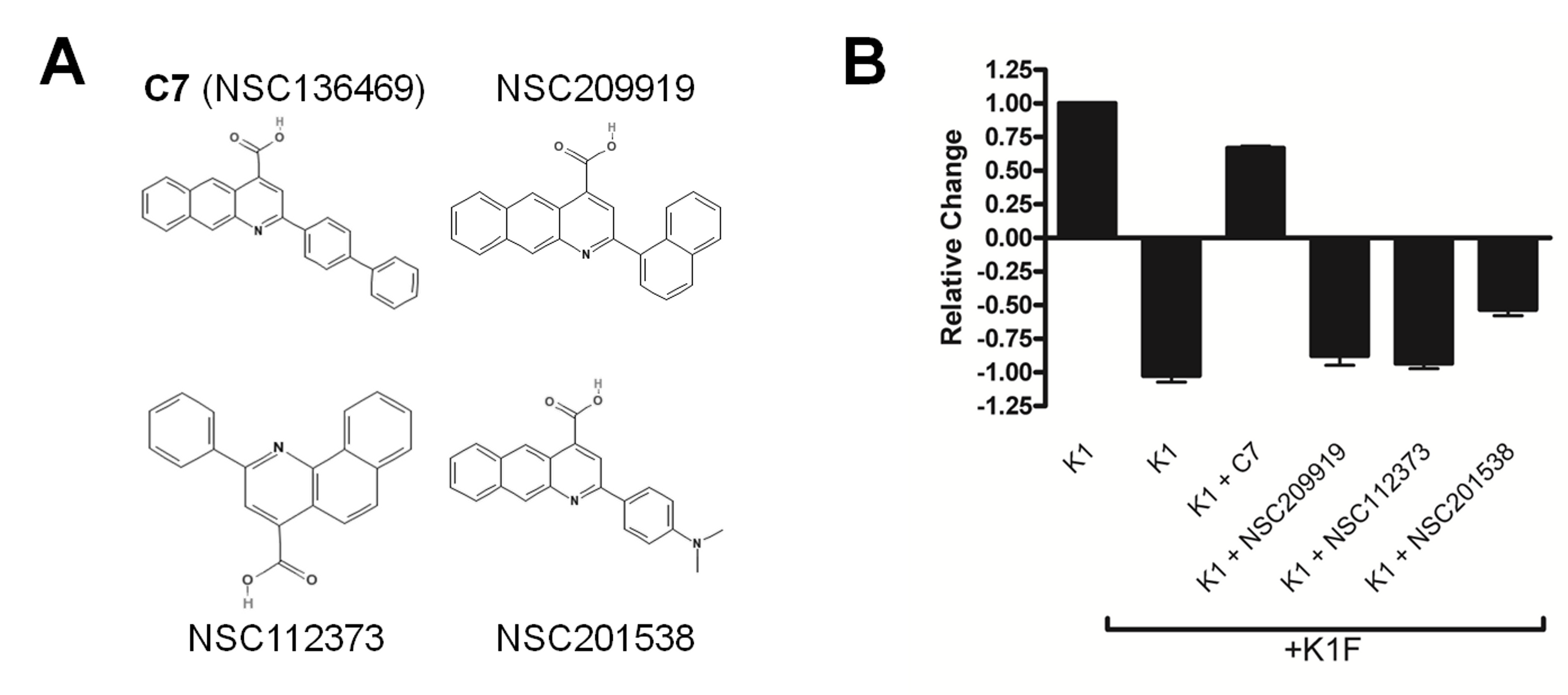

Supplement: Figure S3 — Compounds similar to C7 do not inhibit K1 capsule-dependent phage lysis. Panel A: Analogues of C7 tested as K1 capsule biogenesis inhibitors. Panel B: K1F phage sensitivity assays with 100 µM C7 and analogues. (0.98 MB TIF) [file pone.0011642.s004.tif]
