# Supplementary material for: High-Throughput Identification of Chemical Inhibitors of E. coli Group 2 Capsule Biogenesis as Anti-Virulence Agents
Source: PLoS One. 2010 Jul 19;5(7):e11642. doi: 10.1371/journal.pone.0011642 (PMC2906519; doi:10.1371/journal.pone.0011642)
Supplement: Table S1 — Similarity and identity of key proteins of group 2 capsule biogenesis in E. coli. UTI89 (K1, Group 2 capsule) proteins were compared to other E. coli Group 2 capsule homologues and minimum percent identity and similarity indicated. The Basic Local Alignment Search Tool (BLAST, NCBI) was used to compare key Group 2 capsule assembly proteins from the prototypic K1 strain UTI89 with sequenced E. coli genomes (taxonomic ID 562) with Group 2 capsule gene arrangement. The following sequenced Escherichia coli genomes were considered in the BLAST search: UTI89, SMS 3-5, ED1a, IAI39, APEC01, S88, 042, F11, SE15, BL21 (DE3), Nissle 1917, 101-1. (0.03 MB DOC) [file pone.0011642.s005.doc]

| **UTI89 accession #** | **Protein** | **% identity** | **% similarity** |
| --- | --- | --- | --- |
| YP_542342.1 | KpsD | 97 | 99 |
| YP_542343.1 | KpsU | 94 | 95 |
| YP_542344.1 | KpsC | 96 | 98 |
| YP_542345.1 | KpsS | 98 | 98 |
|  |  |  |  |
| YP_542352.1 | KpsT | 70 | 84 |
| YP_542353.1 | KpsM | 97 | 98 |
